# Functional lignocellulosic materials prepared by ATRP from a wood scaffold

**DOI:** 10.1038/srep31287

**Published:** 2016-08-10

**Authors:** Etienne Cabane, Tobias Keplinger, Tina Künniger, Vivian Merk, Ingo Burgert

**Affiliations:** 1 Wood Materials Science, ETH Zürich, Stefano-Franscini-Platz 3, CH-8093 Zürich, Switzerland; 2 Applied Wood Materials, EMPA – Swiss Federal Laboratories for Materials Science and Technology, Überlandstrasse 129, CH-8600 Dübendorf, Switzerland

## Abstract

Wood, a natural and abundant source of organic polymers, has been used as a scaffold to develop novel wood-polymer hybrid materials. Through a two-step surface-initiated Atom Transfer Radical Polymerization (ATRP), the porous wood structure can be effectively modified with polymer chains of various nature. In the present study, polystyrene and poly(N-isopropylacrylamide) were used. As shown with various characterization techniques including confocal Raman microscopy, FTIR, and SEM/EDX, the native wood ultrastructure and features are retained and the polymer chains can be introduced deep within the wood, i.e. inside the wood cell walls. The physical properties of the new materials have been studied, and results indicate that the insertion of polymer chains inside the wood cell wall alters the intrinsic properties of wood to yield a hybrid composite material with new functionalities. This approach to the functionalization of wood could lead to the fabrication of a new class of interesting functional materials and promote innovative utilizations of the renewable resource wood.

Nature offers a multitude of examples of biological materials having complex hierarchical structures that are nowadays inspiring materials scientists in their quest for new designs and processes^1^,^2^,^3^,^4^,^5^,^6^. In recent years, bioinspiration from marine organisms, insects, or plant tissues has led to significant findings in fields as diverse as mechanical reinforcement of composites, photonics, wettability of surfaces, or self-healing^2^,^7^,^8^,^9^,^10^. To develop such materials mimicking the structural and functional features found in biological materials, a few manufacturing techniques are currently used, including but not limited to polymeric foam templating, freeze casting, braiding pultrusion, and additive manufacturing technologies, the most promising of which are 3D-printing approaches^10^,^11^,^12^,^13^.

However, despite the possibilities and the promising studies reporting on the fabrication of bio-inspired materials with well-defined ultrastructures, major limitations have yet to be overcome^2^,^14^. Among important challenges, conventional fabrication techniques are energy-intensive, they are still far from achieving the extraordinary complexity and functionality of biological materials, and finally, the replication of nano- and micro-features across multiple length scales remains very difficult to achieve. As opposed to the conventional bottom-up approaches, the utilization of readily available biological materials should facilitate the fabrication of nano- and micro-structured solids: as an example, wood already provides the intrinsic solution to avoid high energy expenditures, to reach a high level of complexity, as well as to transfer nanostructural functionalization to larger scales^15^. This is accompanied by exceptional mechanical properties with regard to its lightweight, based on a nano-fiber composite of the cell walls and the unique hierarchical structure of wood across multiple length scales.

At the macromolecular scale, wood cell walls are composed of oriented cellulose nanofibrils (typically 2.5–3.5 nm in diameter), bundled together to form larger cellulose fibril aggregates (roughly 20 nm in diameter) and embedded within an amorphous matrix composed of lignin and hemicelluloses^16^,^17^. In the living tree, wood is not only grown to provide mechanical strength, but it is also designed to transport fluids and nutrients over several meters. These features are reflected at the next hierarchical level, where, in terms of softwoods, the wood structure is characterized by the distribution of the wood tracheids, i.e. hollow tube-like structures with various biological functionalities, geometries, and sizes – typically of around 3 mm length and a diameter between 10–50µm, depending whether latewood or earlywood is considered – bundled together to form a cellular composite material^18^. Providing that its structural features are preserved, the direct utilization of wood as a hierarchically ordered scaffold might be very useful for a wide range of potential applications. This is perhaps best shown by recent pioneering works that are now drawing attention to the potential of wood for novel applications^19^,^20^,^21^,^22^,^23^,^24^, and the trend will likely develop in the future, where the concern towards the use of bio-based resources will become even more crucial.

The wood structure as a biological template to produce biomorphic ceramic scaffolds has already been shown^25^,^26^. In this top-down approach, the wood nano- and micro-features are essentially preserved^27^,^28^. However, the bio-templating processes have led to materials with altered mechanical properties, when compared to the initial wood material. The alterations relate in particular to strength and toughness behaviors: ceramics are typically brittle and hence biomorphic ceramics materials may be challenging to use for certain applications. More importantly, possibilities for post-modifications to bring functionality to the ceramic scaffolds are limited.

Hence, the utilization of solid wood as the basis for advanced materials requires new concepts and alternative methods that could both preserve the integral bulk wood structure and its native properties, and at the same time introduce functionality inside it. These would be extremely useful to develop a completely new class of hybrid materials based on the natural cellulose/lignin wood composite, but with enhanced or novel properties.

We propose to fulfill these two requirements – wood structure preservation and functionalization – by combining the native wood material with grafted polymer chains inside the tracheids cell walls. In this article, we report on the *in-situ* polymerization of styrene and N-Isopropylacrylamide within the native wood structure using Atom Transfer Radical Polymerization (ATRP) for the first time. ATRP is a robust and versatile polymerization technique offering excellent control over polymer chain length and polydispersity^29^. and it can be used to graft polymer brushes to various organic and inorganic materials^30^. It has been used previously to grow functional polymers on isolated cellulose fibers^31^, lignin^32^, as well as at the wood surface^33^.

It should be emphasized that our work bridges two very important research areas related to wood and wood-based materials. On the one hand, our synthesis approach is closely associated to recent activities and efforts developed in the field of nanocellulose-based materials with the use of advanced grafting chemistries, but it is different since we retain and make beneficial use of the wood microstructure. On the other hand, while we perform a chemical modification on bulk wood, our goal differs from classical wood modifications aiming at wood protection and/or dimensional stability, because our goal is to bring new functionalities into wood. Recently, two groups simultaneously reported on the fabrication of “transparent wood”, where the wood scaffold is retained but chemically modified to achieve transparency: these studies demonstrate a very similar philosophy^34^,^35^.

Herein, we show how the newly added polymer chains at the cell wall level (i.e. macromolecular level) can change the intrinsic properties of the cell wall, and result in a wood-polymer hybrid material. We also demonstrate that the functionality of the synthetic polymers used can be transferred to the bulk wood material, and yield functional lignocellulosic materials with novel property profiles that have not been reported before. An important parameter of this wood functionalization targeting the cell wall is the transport of the reactive species inside the solid wood with the use of mild conditions preserving the native wood structure. After the *in situ* polymerization is performed, a careful characterization by means of advanced techniques such as confocal Raman microscopy is required to reveal the distribution of the newly added material within the wood scaffold.

## Results and Discussion

### Polymerization within the wood structure

**Figure 1:**
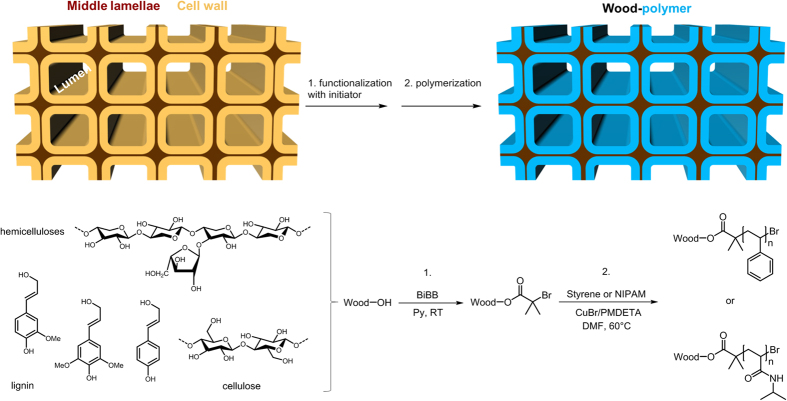
Scheme depicting the basic concept of grafting polymer chains within the bulk wood structure using ATRP. In the first step, the wood scaffold composed of lignin, cellulose and hemicellulose polymers is functionalized with α-bromoisobutyryl bromide (BiBB). In the second step, polymer chains are grown from the attached BiBB initiator molecules in the cell wall, using classical ATRP conditions.

To obtain novel wood-based materials, we prepared spruce (*Picea abies*) wood samples according to a two-step process described in Fig. 1 (full experimental conditions are given in experimental section).

In the first step, we treated wood samples with α-bromoisobutyril bromide (BiBB). In this reaction, the hydroxyl functionalities of the wood natural components are esterified, and the attached brominated compound in the wood scaffold yields a solid macroinitiator for ATRP (denoted as W-Br). The amount of the halide compound attached to wood can be tuned, according to the reaction time used, the concentration, the stoichiometry, and to the ability of the solvent to swell the wood structure (see Supplementary Information, Figure S1). The resulting wood samples obtained after the esterification gain from 5 to 35% weight (given as weight percent gain, WPG, see equation in Supplementary Information) for the reactions performed in THF/Et_3_N and pyridine solutions respectively. This shows that the choice of a good solvent for wood^36^, such as pyridine, favors the penetration of small molecules deep inside the cell wall structure. In the second step, we performed *in situ* polymerization, using W-Br as the ATRP macroinitiator. Styrene (St) and N-Isopropylacrylamide (NIPAM) were chosen as monomers, and a copper-based complex CuBr/N,N,N′,N′,N′′-pentamethyldiethylenetriamine (PMDETA) as catalyst for both styrenic and acrylic monomers. The ATRP ratio ([Monomer]:[Initiator]:[Catalyst]:[Ligand]) was finally set to 50:1:1:3 for both polymerizations, based on the amount of brominated initiator attached to wood (see Supplementary Information for calculations). The amount of added polymer was easily estimated from the weight gain after reaction completion.

### Characterization of wood-polymer hybrids

**Figure 2:**
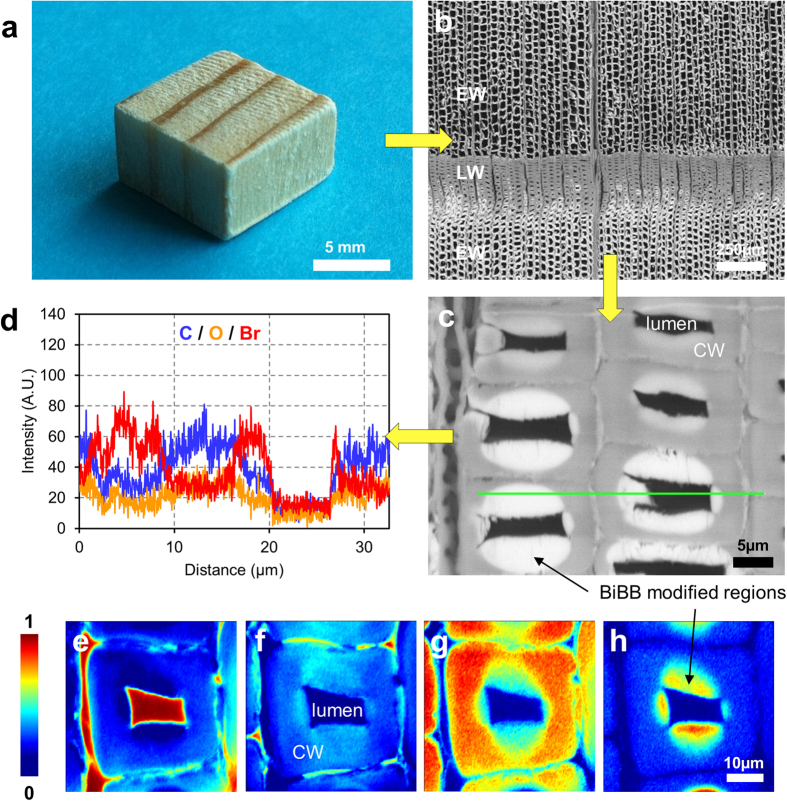
Detailed characterization of wood modified with ATRP initiator (W-Br). (**a**) photograph of a spruce wood sample. (**b,c**) SEM images of a W-Br cube at different magnifications showing the preserved structure of wood after functionalization (EW: early wood, LW: late wood, CW: cell wall), and showing the EDX profile line. (**d**) Distribution of cell wall elements (carbon and oxygen), and bromine (from the BiBB molecules) across the profile drawn in (**c**). (**e–h**) Example of VCA analysis on a wood cross-section, revealing the distribution of the BiBB molecule deep within a LW cell wall. From left to right: background signal (**e**), lignin rich regions (**f**), cellulose rich region (**g**), BiBB-modified regions (**h**).

We confirmed the successful esterification of wood with FTIR spectroscopy, presented in the Supplementary Information (Figure S2). Although the C=O peak is present in native wood, as part of the lignin polymer, the W-Br spectra obtained reveal an increased intensity of the ester C=O bond (1735 cm^−1^), correlated with a slight decrease in the broad OH signal (3350 cm^−1^) after reaction. In addition, the presence of the BiBB molecule in wood is shown by the small peak assigned to the C-Br stretch at 753 cm^−1^. We also performed EDX and confocal Raman microscopy measurements in order to visualize the spatial distribution of the halogenated compound in wood after the first step of the functionalization (Fig. 2). EDX profiles reveal the presence of bromine across the entire cell wall depth in the early wood regions (thin cell walls, ca. 2 μm thick), and the intensity profiles for the brominated compound presented in the Supplementary Information correlate well with the calculated weight gains (Figure S3). A more gradual and inhomogeneous distribution was observed for the late wood regions where the cell walls are thicker (up to 10 μm thick) (Fig. 2d). We observed this distribution pattern in previous studies conducted with other molecules^37^,^38^,^39^, and this is an indication that the penetration of reactive species from the lumen to the cell wall is limited by diffusion.

Finally, as shown in Fig. 2e–h, we applied Raman spectroscopy on the wood cross-sections to demonstrate the presence of the newly attached BiBB molecule into the wood structure. In order to help visualizing regions with different chemical compositions in the modified wood samples, vertex component analysis (VCA) was applied to the spectral dataset obtained. This method is highly advantageous in imaging spectroscopy to deconvolute highly complex spectra. For a detailed description of the technique, the reader is referred to the work from Nascimento *et al*.^40^. Figure 2f,g respectively reveal the distribution of lignin rich regions (cell corners), and cellulose rich regions (cell wall), while Fig. 2e shows the background, i.e. lumen area. As observed with the EDX profiles, Raman images confirm penetration to a depth of up to 5 μm in the thick-walled latewood fibers (Fig. 2h).

For polystyrene and PNIPAM modified wood samples (respectively W-PSt and W-PNIPAM), the final amount of polymer added can be determined independently from the first weight gain (see Supplementary Information, Table S1). As shown in Supplementary Information (Figure S4), changing the solvent while keeping other conditions constant significantly influenced the final polymer gain. Finally, even though it is not considered as an ideal solvent for ATRP reactions^41^, both polymerizations were conducted at 60 °C in dimethylformamide (DMF). For the NIPAM reaction, commonly used DMF/water mixtures (or other aqueous media) did not prove successful with our W-Br macroinitiator: the esterification of wood with BiBB significantly reduces the amount of hydroxyl groups accessible in the cell wall, and as a result, the W-Br samples are slightly hydrophobic. An aqueous ATRP solvent mixture therefore has limited swelling capability when compared to pure DMF.

**Figure 3:**
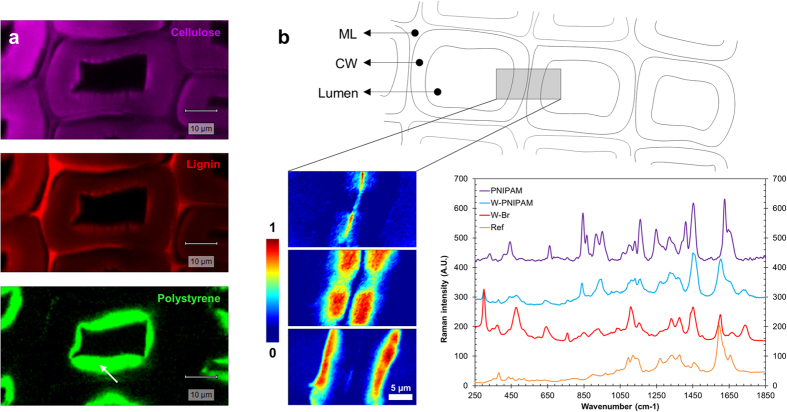
Distribution of synthetic polymer chains within the wood structure, as observed with confocal Raman microscopy for W-PSt and W-PNIPAM. (**a**) natural polymer and polystyrene signals after integration of the specific bands (integration windows for cellulose: 1068–1190 cm^−1^; lignin: 1550–1700 cm^−1^; PSt: 968–1020 cm^−1^). (**b**) distribution of the PNIPAM polymer obtained through VCA method (from top to bottom: lignin-rich middle lamellae (ML), cell wall region dominated by the cellulose signal, and cell wall region dominated by the PNIPAM signal). The graph shows representative spectra of the various components studied by Raman spectroscopy.

FTIR spectra of the wood-polymer hybrids are given in Supplementary Information (Figure S5 and S6). They indicate the presence of the expected polymers in wood, and confirm the different weight gains calculated (see Table S1 in Supplementary Information). Confocal Raman microscopy was also employed to assess the presence of polymers following ATRP in wood, and to provide images of their distribution. As shown in Fig. 3a, newly added polystyrene chains can be evidenced in the cell wall. In the case of W-PSt samples, a simple integration of the peak assigned to phenyl ring vibrations was sufficient to obtain an adequate mapping, since the polystyrene spectral signature is easily distinct from cell wall natural polymers signals. In the case of W-PNIPAM, we obtained Raman images through the VCA method.

Early wood, transition wood, and late wood areas were analyzed, however the scanning area was limited to large profiles including two adjacent cell walls. According to the images shown in Fig. 3b, the polymer chains were successfully found inside the cell wall, at a depth up to 5–6 μm, and their distribution pattern reflects the distribution of the initiator.

Further earlywood and latewood images of modified spruce can be seen in Supplementary Information (Figure S7). The obvious collocation of the ATRP initiator with the polymer chains (as observed with Raman imaging) associated to the stability of the added polymers within the cell wall upon washing with various solvents, constitute an indirect proof of the successful polymer grafting.

### Physical properties of wood-polymer hybrid materials

Due to the nature and the arrangement of the polymers constituting the cell wall of tracheids, wood has a characteristic hygroresponsive behavior that can easily be observed via changes in both weight (due to water uptake or loss) and dimensions (due to cell wall swelling or shrinking) upon fluctuations in environmental conditions (humidity)^42^. This low dimensional stability may become a severe drawback for the utilization of wood in various applications, where reliable materials are needed. To minimize the cell wall water uptake, several approaches to the chemical alterations of the wood cell wall components have been developed^43^. Essentially, the modification techniques for bulk wood, such as acetylation, address the hydroxyl groups in wood, and aim at transforming the hydrophilic nature of wood to a more hydrophobic material. It should be noted that a number of studies report on the polymerization of various monomers inside solid wood. In general, most attempts to introduce hydrophobic monomers (such as styrene or methyl methacrylate) inside the cell wall structure are not efficient, due to poor penetration, and lead to lumen filling exclusively^44^,^45^,^46^. A few recent studies have shown that more hydrophilic compounds (such as hydroxyethyl methacrylate and acrylated poly(ethylene glycol)) can be successfully polymerized in both the cell walls and the lumina, leading to modified wood with enhanced mechanical properties and improved dimensional stability^47^,^48^.

In this study, we chose to work with PSt and PNIPAM because they have properties potentially altering the hygroscopic nature of wood, and by using a cell wall initiated polymerization, we target the functionalization of the cell wall region only. Polystyrene is a hydrophobic polymer that, if present in the cell walls, should reduce the water uptake and consequently limit wood swelling^19^,^37^,^39^. More interestingly, PNIPAM displays a well-known change in microstructure triggered by temperature, switching from hydrophilic to hydrophobic states in a reversible manner^49^, which could even be used to control the wood swelling behavior.

**Figure 4:**
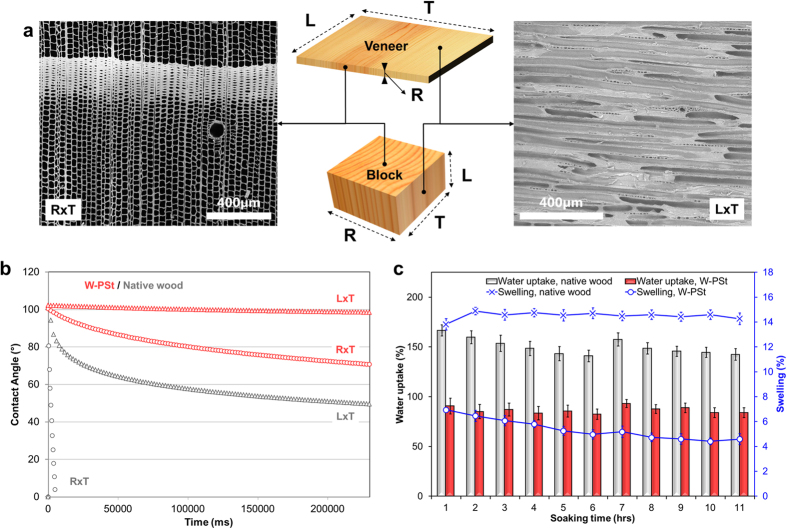
Physical properties of wood grafted with polystyrene. (**a**) scheme illustrating the different wood surfaces tested for wettability measurements, according to the longitudinal (L), radial (R), and tangential (T) directions. (**b**) surface properties as seen with contact angle measurements on the L × T and R × T faces, for native wood and W-PSt (each curve is averaged over three measurements, lower and upper limits are given in Figure S8). (**c**) water uptake and swelling behaviour as a function of time, for untreated and PSt-grafted wood cubes.

Therefore, to study the effect of the polymer chains grafted in the cell wall structure on the new materials properties, we investigated the wood-water interactions of W-PSt and W-PNIPAM samples. In addition, because wood is a highly anisotropic material, we considered the various surfaces of our wood samples, as well as wood as a bulk material, to provide an exhaustive overview of these properties (see Fig. 4a for clarification).

At the L × T wood surface, the open fiber structure is now decorated with polymer brushes. In the case of the grafting of polystyrene chains in wood, we observe a clear hydrophobic effect, due to both the diminution of OH functionalities at the surface and to the polymer chains themselves (Supplementary Information, scheme S1). The contact angle measurements given in Fig. 4b, show a clear wettability difference between the native wood and the PSt-grafted L × T surfaces.

In the case of the R × T surfaces (cross-section perpendicular to the fiber direction), we observed an even more pronounced effect. With the R × T surface, the water penetration in a native wood cube is accelerated by a strong capillary effect and water is immediately soaked inside. Our measurements show that the contact angle on the R × T face of a W-PSt cube is comparable with the value obtained for the L × T face. The PSt chains attached at the lumen interface and the built-in modification provided by the functionalization at the cell wall level clearly retard or suppress the capillary effect, resulting in a rather hydrophobic surface.

The effect observed at the surface, is therefore reflecting the successful introduction of polystyrene chains into the wood structure. The cell wall hydrophobic media (after esterification of the OH functionalities, and bulking effect from the polystyrene chains), reduces water penetration into wood, as illustrated by the significant differences in water uptake shown in Fig. 4c (almost 50% reduction). One can also clearly observe that a reduced water uptake is directly related to a limited swelling of the wood structure. Because the access of water molecules to the cell wall is reduced, the total swelling is reduced by more than 65% after 11 hours of water immersion (a more detailed dimensional stability study for W-PSt samples is given in supplementary information, in Figures S9 and S10).

**Figure 5:**
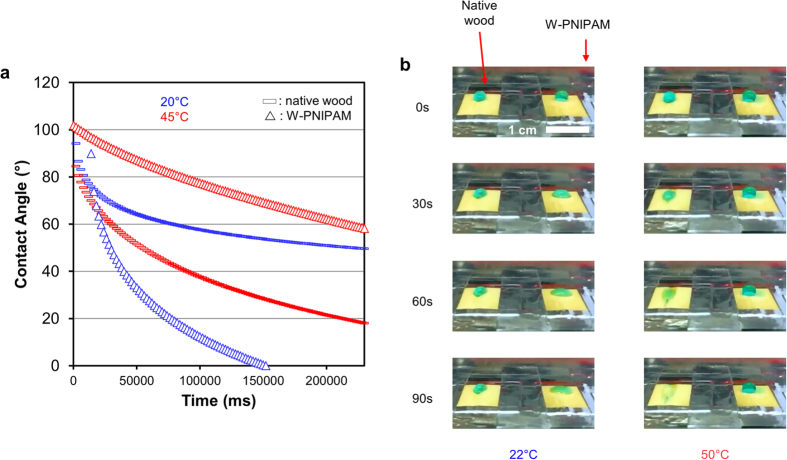
Surface properties of native and PNIPAM modified spruce wood veneers (L × T). (**a**) Contact angle measurements for native wood surfaces and W-PNIPAM samples at 20 °C and at 45 °C in a temperature controlled chamber (each curve is averaged over five measurements, lower and upper limits are given in Figure S8). (**b**) Photographs of spruce veneers (0.5 mm thickness), placed on a heating plate, and showing the evolution of a water droplet (dyed with neolan glaucin E–A) on the surface of wood, according to temperature (22 °C corresponds to the standard lab conditions, and 50 °C corresponds to the lowest controlled temperature achieved with the heating plate).

As shown in Fig. 5, the W-PNIPAM surfaces (L × T) exhibited a clear temperature-responsive wetting behavior. These samples showed high wettability at room temperature, i.e. below the Lower Critical Solution Temperature (LCST) of PNIPAM chains (32 °C), even higher than native wood. At higher temperature, drops deposited on the native wood surface are rapidly adsorbed and wet the wood, while the drops placed on the W-PNIPAM surfaces show a much slower subsidence. The temperature-dependent wettability is clearly shown by the contact angle measurements at 20 °C and 45 °C. According to these observations, we show that the surface of wood can be made responsive through the grafting of functional polymers.

**Figure 6:**
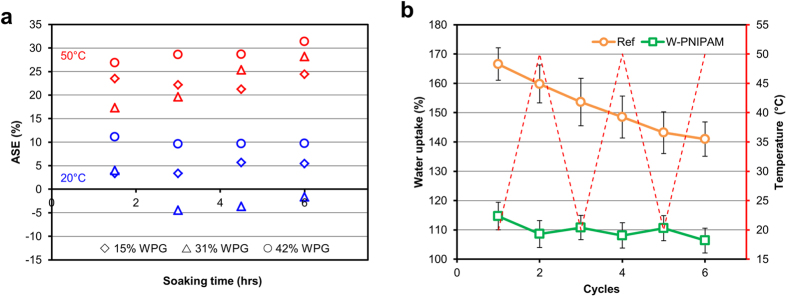
Water uptake and swelling behaviour of W-PNIPAM, highlighting the effect of PNIPAM grafting in the cell wall structure on the bulk properties of the new wood-polymer hybrid material. (**a**) The Anti-Swelling Efficiency is plotted against soaking time in pure water (diamonds: WPG = 15%, triangles: WPG = 31%, and circles: WPG = 42%). (**b**) Graphic showing the evolution of water uptake upon several temperature cycles (20 to 50 °C), for both native wood and W-PNIPAM samples.

Besides the interesting surface effect obtained, the grafting of PNIPAM polymer chains from the cell wall components should transform the wood into a responsive bulk material (see Supplementary Information, Scheme S1). When a PNIPAM modified wood cube is placed in water at room temperature, we observe a total volumetric swelling around 12.5%, which is comparable to a reference spruce wood sample. At higher temperatures (50 °C), above the LCST of PNIPAM (32 °C), the total volumetric swelling of the W-PNIPAM cubes reaches 10%, which represents a significant decrease. These results are shown in Fig. 6a, where we plotted the Anti-Swelling Efficiency (ASE) for wood blocks with various WPGs and at two temperatures (equation for ASE is given in Supplementary Information). ASE represents the swelling difference between a reference spruce wood sample and the W-PNIPAM samples. At room temperature, ASE values range from −5 to 10%, indicating that the W-PNIPAM samples behave almost like native wood samples (i.e. their ability to take up water and to swell is not affected). On the contrary, above the PNIPAM LCST, hot water was not able to penetrate into the cell wall, displaying a hydrophobic nature due to the collapsed conformation of the PNIPAM chains, and a 25–30% reduction in total swelling was obtained. Therefore these W-PNIPAM cubes show a temperature dependent swelling, which implies that by addressing the cell wall nature through the grafting of a functional polymer, the intrinsic properties of the initial wood material were effectively altered.

Bearing in mind the promising results obtained with the W-PNIPAM material in terms of swelling, we took a step forward to explore the reversibility of the responsive behavior. In a typical experiment, we placed W-PNIPAM cubes in water and we applied temperature cycles from 20 °C to 50 °C. We then observed the evolution of the water gain in the material, after an equilibration time of two hours. As shown in Fig. 6b, we observed a rather limited, but nonetheless clear temperature dependence of the water uptake for the W-PNIPAM samples: at 20 °C, the total water uptake is systematically higher than at 50 °C, which is again a proof that our hybrid materials display new temperature-responsive properties after grafting of PNIPAM chains.

## Conclusions

We have prepared hybrid wood-polymer materials with polymer chains synthesized within the cell wall of solid wood samples with a two-step process based on ATRP, and we have demonstrated how these grafted polymer chains can truly change the intrinsic properties of wood. The polymer chains are grown directly from the modified cell walls, in the preserved wood hierarchical structure, and the amount and distribution of the added polymer in the wood scaffold can be tuned. The result is a completely new hybrid material, having the exceptional structural features of wood, with built-in new functionalities, according to the nature of the grafted polymer chains. The resulting W-PSt materials show a convincing hydrophobic character, both at the surface and as a bulk property. By choosing a functional monomer, N-Isopropylacrylamide, we are able to transfer a temperature controlled response in terms of dimensional change and water uptake to an otherwise rather temperature passive material. The possibility to modify different wood scaffolds with functional monomers in a controlled manner represents a real progress and will lead to the fabrication of advanced functional materials with important benefits: our functionalization approach, coupled with the intrinsic advantages of lignocellulosic materials, (i.e. sustainability, easy access to large scales, micro-scale anisotropic porous structure) yields novel wood-polymer hybrid materials that could find applications in the future in very diverse areas, such as scaffolds for filters and membranes, large façade elements for low energy buildings, or actuation materials for sensors.

Hence, the functionalization process may allow for capturing new fields of application for the renewable resource wood and for substituting less eco-friendly materials. Nonetheless, we are aware that in its present form the functionalization protocol is not adapted to the exigencies related to up-scaling challenges, in particular in terms of green chemistry. We are therefore focusing on solutions, in order to lower the amount of catalyst by investigating the feasibility of the ICAR and ARGET ATRP techniques on wood^50^. The optimization of wood sample geometries and cleaning procedures can lead to a reduced amount of solvents in the process. In parallel, we are working on the use of alternative solvents, such as supercritical fluids and ionic liquids, which are considered more environmentally friendly.

## Methods

### Reagents and materials

All reagents and materials were purchased from Sigma-Aldrich and used as received. Wood from Norway spruce (*Picea abies*) was processed to prepare blocks of 10 × 10 × 5 mm^3^, Radial × Tangential × Longitudinal dimensions, and veneers with 0.5 mm thickness, 2 × 2 cm^2^. Before the first functionalization step, the samples were dried in the oven at 65 °C until a constant mass was reached.

### Functionalization of wood with ATRP initiator

Oven dried wood samples were placed under vacuum (10mbar) in a flask caped with a septum. A BiBB solution (either in anhydrous pyridine, anhydrous DCM/Et_3_N, or anhydrous THF/Et_3_N) was then slowly added with a syringe. The amount of BiBB used was calculated as a 0.5 molar equivalent with wood glucopyranose equivalents (MW = 162 g/mol). The reaction system was stirred at room temperature for a given amount of time. The wood cubes were withdrawn, blotted, and washed with acetone to remove unreacted BiBB and amine salts (three times). The functionalized wood obtained (W-Br) was then dried under full vacuum at 65 °C for 24 hours.

### ATRP of styrene and NIPAM

In a typical polymerization, NIPAM was polymerized with W-Br as the macroinitiator, in DMF, using the following concentration ratio: [Monomer]/[W-Br]/[CuBr]/[PMDETA] = 50:1:1:3. W-Br samples were placed in a Schlenk flask equipped with a gas inlet and a septum. In a separate Schlenk flask, we placed CuBr, ligand, monomer, and solvent. The solution was then degassed with 3 freeze-pump-thaw cycles. After complete degassing, the ATRP solution was cannula-transferred to the flask containing the W-Br blocks, and heated to 65 °C. The polymerization was conducted for a given amount of time under tight nitrogen atmosphere. After reaction completion, the flask was opened to air; the wood samples were removed from the flask and washed with several volumes of acetone (or EtOH or water). Finally, the W-Polymer samples were dried under vacuum at 65 °C for 24 hours.

### Characterization

Smooth surfaces of the wood cubes were prepared using a rotary microtome (Leica Ultracut, Germany). Scanning electron microscopy was carried out on a FEI Quanta 200 3D probe in the low-vacuum mode (0.53 Torr) driven with an acceleration voltage of 20 kV. The SEM was equipped with a backscattered electron and a secondary electron detector and an EDAX energy-dispersive X-ray spectrometer. Semi-quantitative information on the elemental distribution and concentration was obtained by EDX line-scanning of the chemically modified cell wall. Fourier transform infrared spectroscopy (FTIR) measurements were conducted on a tensor27 from Bruker instruments, equipped with an ATR module. Spectra were baseline-corrected and smoothed in the OPUS software. Raman spectroscopy and imaging: 15–20 μm thick cross-sections were prepared using a rotary microtome (Leica Ultracut, Germany). The cross-sections were placed on a glass slide in wet conditions (deuterium oxide) and sealed under a cover slip. For further details on the sample preparation the reader is referred to the work by Gierlinger *et al*.^51^. The measurements of the samples were performed with a confocal Raman microscope (Renishaw InVia, Wotton-under-Edge, England) using a 532 nm laser, an oil immersion objective (Nikon, 100x, NA = 1.3, 0.17 mm coverslip corrected) and a 1800 l/mm grating. As mapping parameters, an integration time between 0.15 s and 0.20 s and a step width of 300 nm were used. For the VCA, the map data were exported into CytoSpec, a commercially available MatLab-based software. Contact Angle (CA) measurements: The cross-sections of the samples were smoothed with a sledge microtome GSL 1 (WSL, Birmensdorf, Switzerland). All samples were conditioned at 20 °C and 65% RH. The static sessile drop method was used to determine the contact angle of water on the different wood surfaces with a Dataphysics OCA-20 contact angle analyser. The measurements were performed at 20 °C and 45 °C in a temperature controlled chamber at a relative humidity of 65%. Deionized distilled water (LiChrosov^®^) droplets of 8 μl were placed onto the wood surface and images were taken maximum 300 seconds at a frame rate of 5 images per second. The CA evolution was analysed by using the image analysing software of the contact angle measuring device.

## Additional Information

**How to cite this article**: Cabane, E. *et al*. Functional lignocellulosic materials prepared by ATRP from a wood scaffold. *Sci. Rep*. **6**, 31287; doi: 10.1038/srep31287 (2016).

## Supplementary Material

Supplementary Information
