# Supplementary material for: Functional lignocellulosic materials prepared by ATRP from a wood scaffold
Source: Sci Rep. 2016 Aug 10;6:31287. doi: 10.1038/srep31287 (PMC4978991; doi:10.1038/srep31287)
Supplement: Supplementary Information [file srep31287-s1.pdf]

Supporting Information for:

Functional lignocellulosic materials prepared by ATRP from a wood scaffold

Etienne Cabane,*[,1,2] Tobias Keplinger,[1,2] Tina Künniger,[2] Vivian Merk,[1,2] & Ingo Burgert[1,2]

[1]Wood Materials Science, ETH Zürich, Stefano-Franscini-Platz 3, CH-8093 Zürich, Switzerland.

[2]Applied Wood Materials, EMPA – Swiss Federal Laboratories for Materials Science and Technology, Überlandstrasse 129, CH-8600 Dübendorf, Switzerland.

*Corresponding author: cabanee@ethz.ch

Experimental section

Amount of brominated initiator grafted in wood: The estimated amount of grafted brominated initiator (BiBB) in the wood structure is based on the weight gain determined after the first reaction is completed.

After the reaction of wood with BiBB and before the determination of the weight gain, the wood samples are thoroughly washed (in order to remove unreacted chemicals) and dried until a constant weight is observed, to make sure that no traces of washing solvents are present, and that the weight gained after reaction is solely due to BiBB molecules.

The mass of BiBB attached to wood was calculated as follows:

$$m_{BiBB} = m_t - m_i$$

Where m_t is the mass of the washed and dried treated wood, and m_i is the mass of the dried untreated wood.

With m_{BiBB}, one can calculate the number of moles of BiBB attached to wood:

$$n_{BiBB} = \frac{m_{BiBB}}{M_{BiBB}}$$

Where M_{BiBB} = 149.99 g/mol (which corresponds to the molecular weight of the BiBB molecule with one Bromine atom less).

The ATRP ratio is then based on n_{BiBB}.

Characterization techniques

Weight percent gain (WPG): the various weight gains (after modification step 1 or 2) were calculated as follow:

$$WPG\ (\%) = \frac{(W_2 - W_1)}{W_1} * 100$$

Where W_2 is the weight of the modified wood samples, and W_1 is the weight of the wood samples before the modification step.

The number of moles of BiBB present in solid wood after the first step is calculated from the weight gain values.

Water uptake (WU): the mass change of the wood samples upon soaking in water was calculated using the following equation:

$$WU\ (\%) = \frac{(W_w - W_d)}{W_d} * 100$$

Where W_w is the weight of the water-soaked wood samples, and W_d is the weight of the dried wood samples.

Water-repellent effectiveness (WRE): the WRE of the modified wood samples was calculated using the following equation:

$$WRE\ (\%) = \frac{(WU_2 - WU_1)}{WU_2} * 100$$

Where WU_1 is the water uptake of the treated sample upon soaking in water, and WU_2 is the water uptake of the untreated wood upon soaking in water.

Swelling (S): the swelling of the wood samples upon soaking in water was calculated using the following equation:

$$S\ (\%) = \frac{(V_2 - V_1)}{V_1} * 100$$

Where V_2 is the volume of the water-soaked wood samples, and V_1 is the volume of the dried wood samples.

Anti-Swelling Efficiency (ASE): the ASE of the modified wood samples upon soaking in water was calculated using the following equation:

$$ASE\ (\%) = \frac{(S_2 - S_1)}{S_2} * 100$$

Where S_2 is the swelling of the untreated wood samples, and S_1 is the swelling of the treated wood samples.

Rowell et al. give a comprehensive description of the physical characterizations related to water repellency and dimensional stability of wood.[1]

Results

Step 1: attachment of BiBB to wood samples

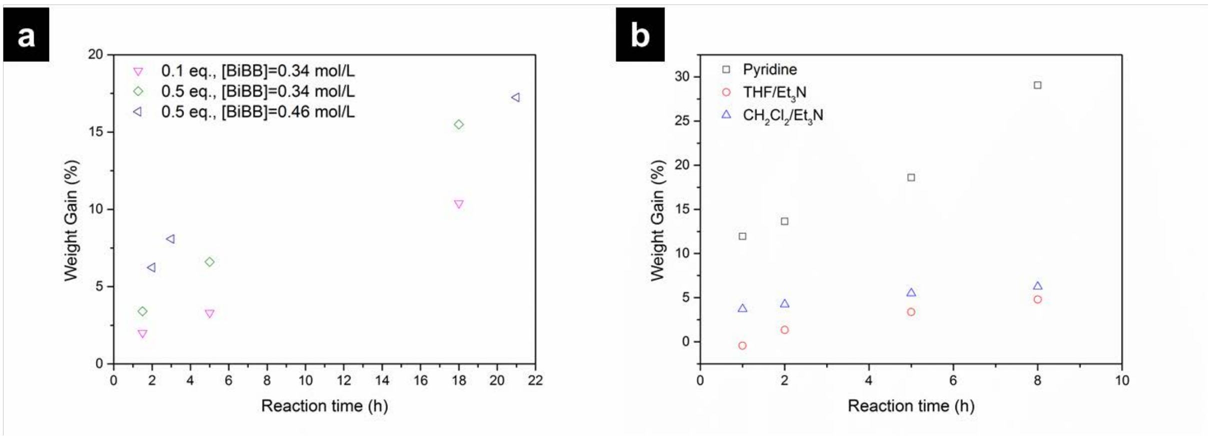

Figure S1: influence of concentration and stoichiometry (a), solvent (b), and reaction time (a and b) on the final initiator weight gain in spruce wood cubes.

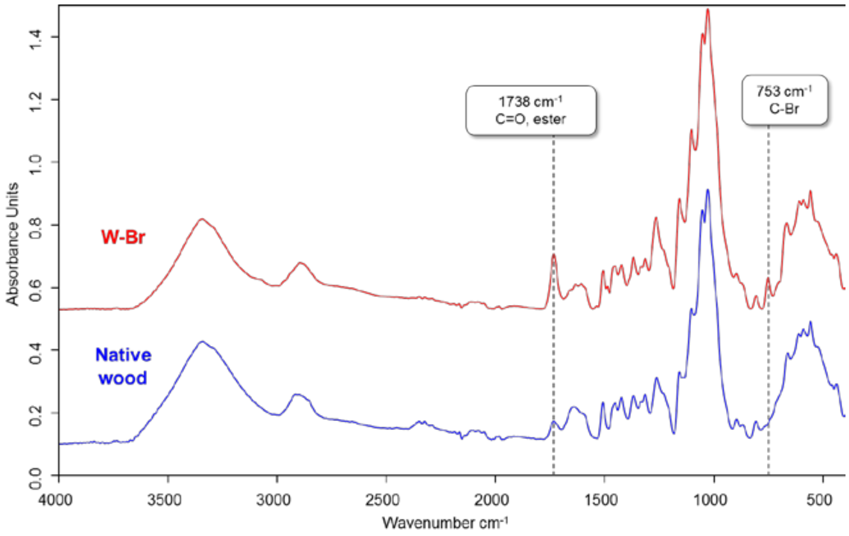

Figure S2: FTIR spectra of native wood, and wood esterified with BiBB, showing the covalent bonding of BiBB to wood.

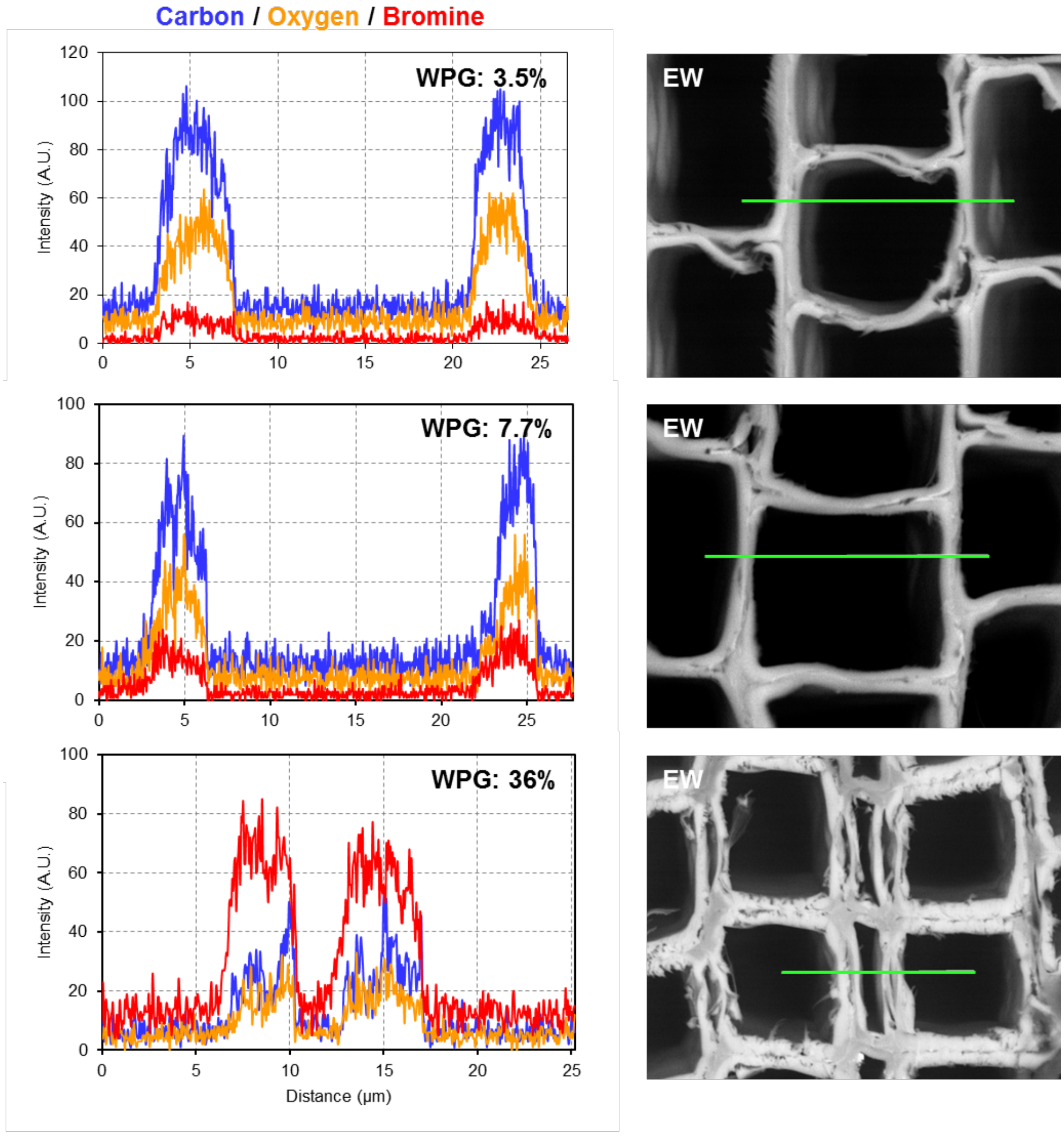

Figure S3: Typical EDX profiles obtained for spruce wood cross-sections (earlywood region) esterified with increasing amounts of α-bromoisobutyril bromide (weight percent gains - WPG). Green lines indicate the profile measured.

Step 2: Polymerization

Table S1: ATRP conditions and various weight gains for W-PSt and W-PNIPAM experiments.

	WPG 1[a] (%)	Monomer	[M]:[I]:[Cu]:[L]	Solvent	Reaction time (h)	[M] (mol/L)	T (°C)	WPG 2[b] (%)	WPG total[c] (%)
Spruce veneers	29.2	NIPAM	100:1:1:1.1	Water/DMF (50:50 v/v)	18	1.91	RT	39.1	79.8
Spruce veneers	29.2	NIPAM	50:1:1:3	IPA	18	0.92	RT	3.3	33.5
Spruce veneers	29.2	NIPAM	50:1:1:3	MeCN	18	0.91	RT	20.2	55.3
Spruce veneers	29.2	NIPAM	50:1:1:3	DMSO	18	0.91	40	43.6	85.6
Spruce veneers	29.2	NIPAM	50:1:1:3	DMF	18	0.91	60	75.8	127.2
Spruce cubes	6.3	NIPAM	100:1:1:1.1	Water/DMF (50:50 v/v)	18	1.02	RT	4.9	12.2
Spruce cubes	13.7	NIPAM	100:1:1:1.1	Water/DMF (50:50 v/v)	18	1.44	RT	1.3	15.5
Spruce cubes	18.6	NIPAM	100:1:1:1.1	Water/DMF (50:50 v/v)	18	2.03	RT	1.1	19.3
Spruce cubes	18.6	NIPAM	100:1:1:1.1	DMF	18	2.00	RT	2.4	20.8
Spruce cubes	3.7	NIPAM	50:1:1:3	DMF	18	0.57	60	4.5	8.4
Spruce cubes	12	NIPAM	50:1:1:3	DMF	18	0.93	60	14.7	28.5
Spruce cubes	23.5	NIPAM	50:1:1:3	DMF	18	0.95	60	42.1	75.5
Spruce cubes	24.1	NIPAM	50:1:1:3	DMF	18	1.04	60	59.3	97.7
Spruce cubes	36	NIPAM	50:1:1:3	DMF	18	0.96	60	31.3	78.6
Spruce cubes	24.1	St	50:1:1:3	DMF	18	1.04	60	102.0	150.7

[a] WPG 1 corresponds to the added amount of BiBB.
[b] WPG 2 corresponds to the added amount of polymer.
[c] WPG total corresponds to the total weight gain (BiBB+polymer).

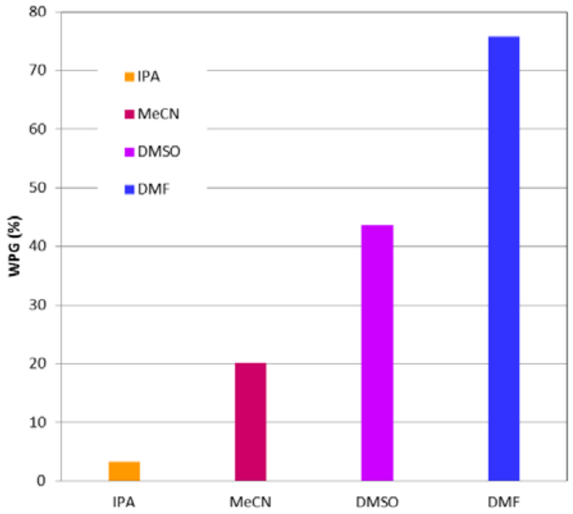

Figure S4: influence of ATRP solvent on the polymer weight gain. ATRP ratio kept constant ([M]:[I]:[Cu]:[L] = 50:1:1:3), reaction temperatures: room temperature for IPA (isopropyl alcohol) and MeCN, 40°C in DMSO and 60°C in DMF.

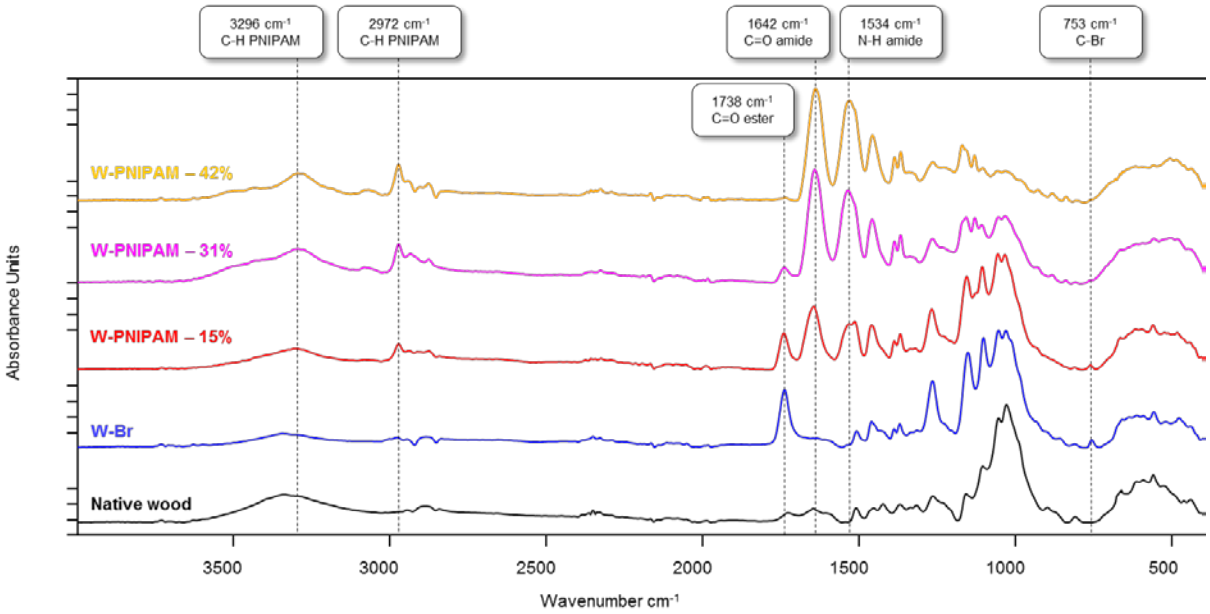

Figure S5: FTIR spectra of reference wood (untreated), wood esterified with ATRP initiator (W-Br), and wood grafted with PNIPAM polymer, with increasing polymer content (W-PNIPAM – X%).

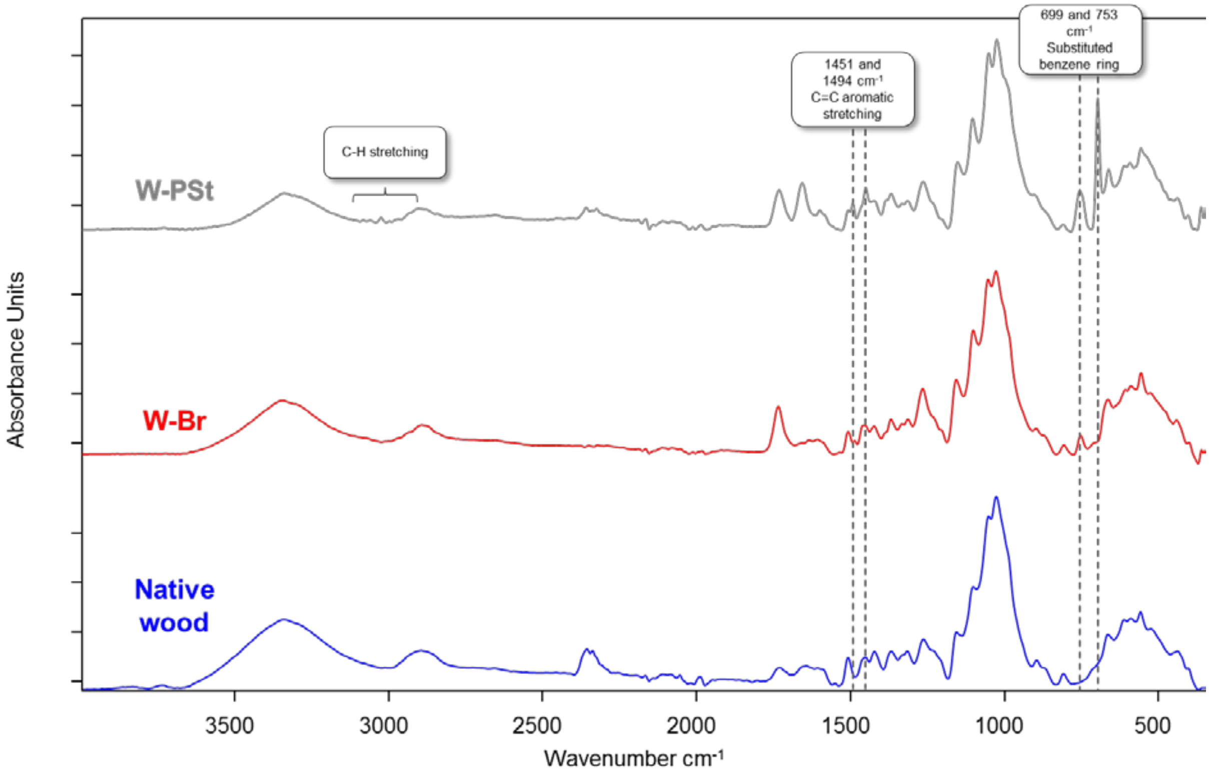

Figure S6: FTIR spectra of native wood (untreated), wood esterified with ATRP initiator (W-Br), and wood grafted with polystyrene polymer (W-PSt).

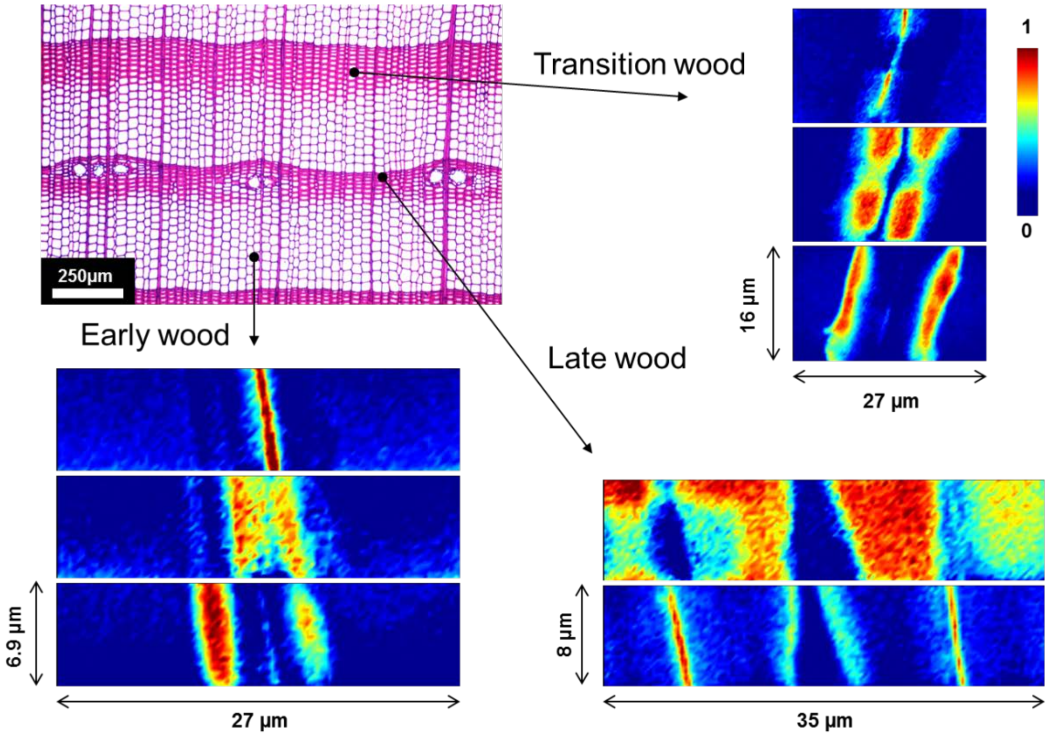

Figure S7: Confocal Raman microscopy images of cell walls grafted with PNIPAM, from late wood, early wood, and transition wood.

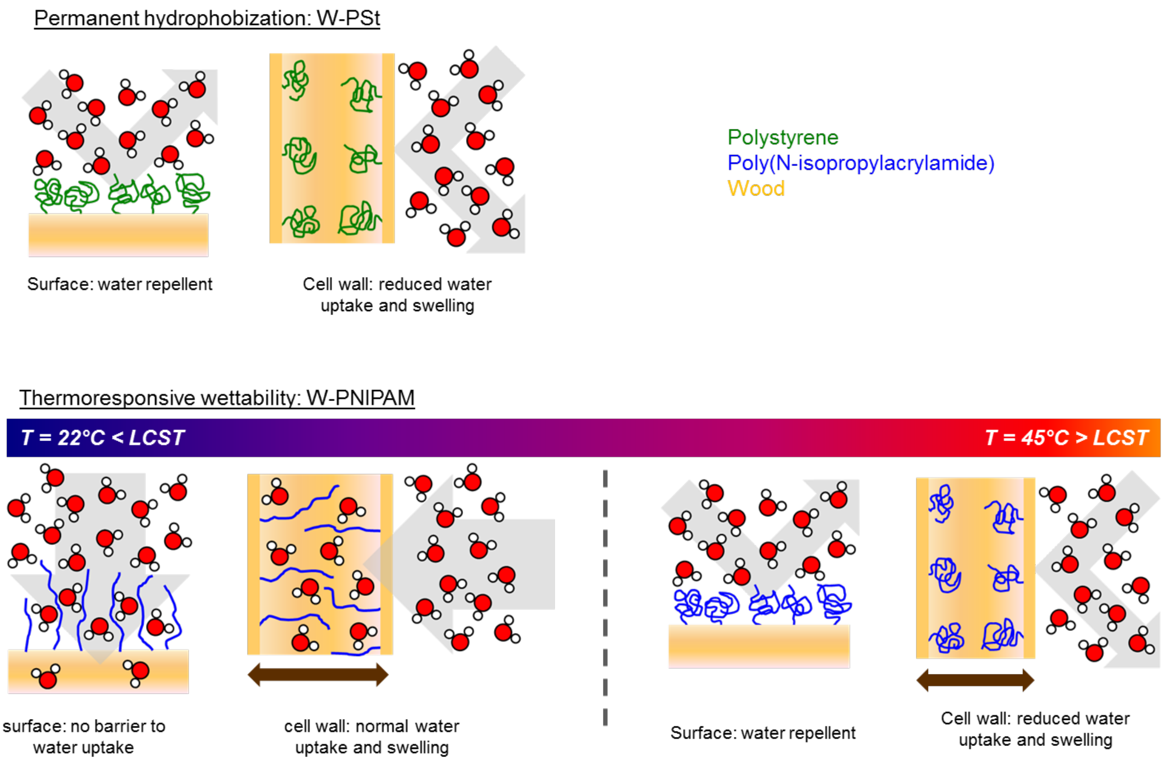

Scheme S1: scheme describing the expected surface and bulk behaviour of wood grafted with polystyrene and poly(n-isopropylacrylamide).

<u>Details for contact angle measurements</u>

a)

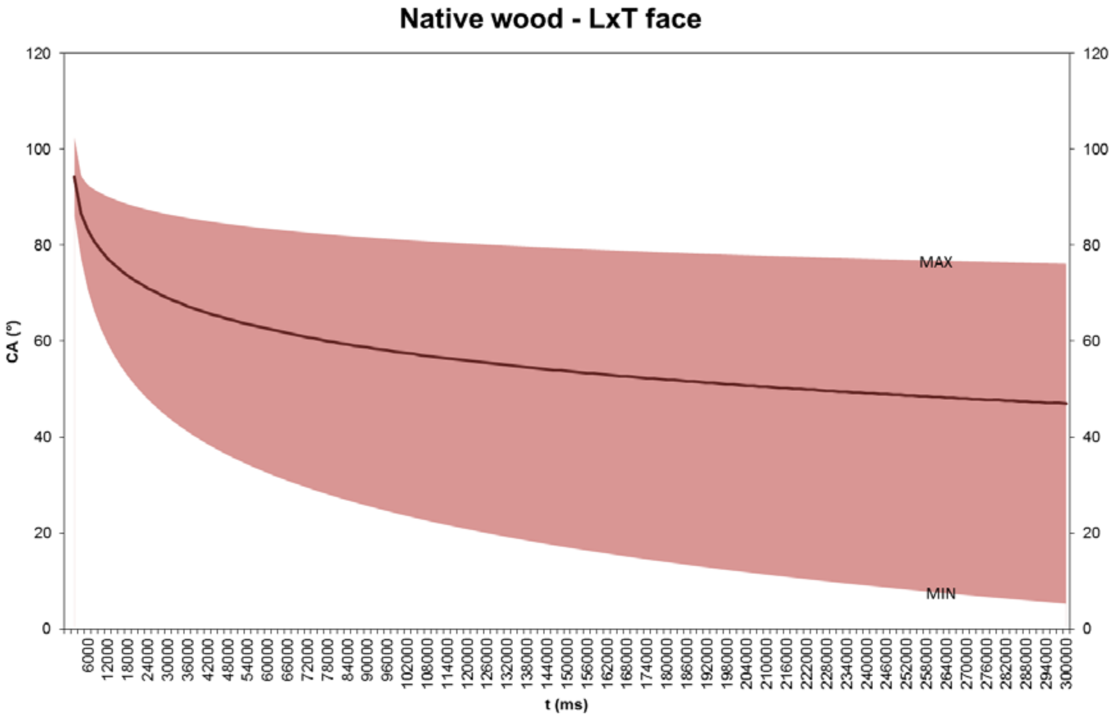

b)

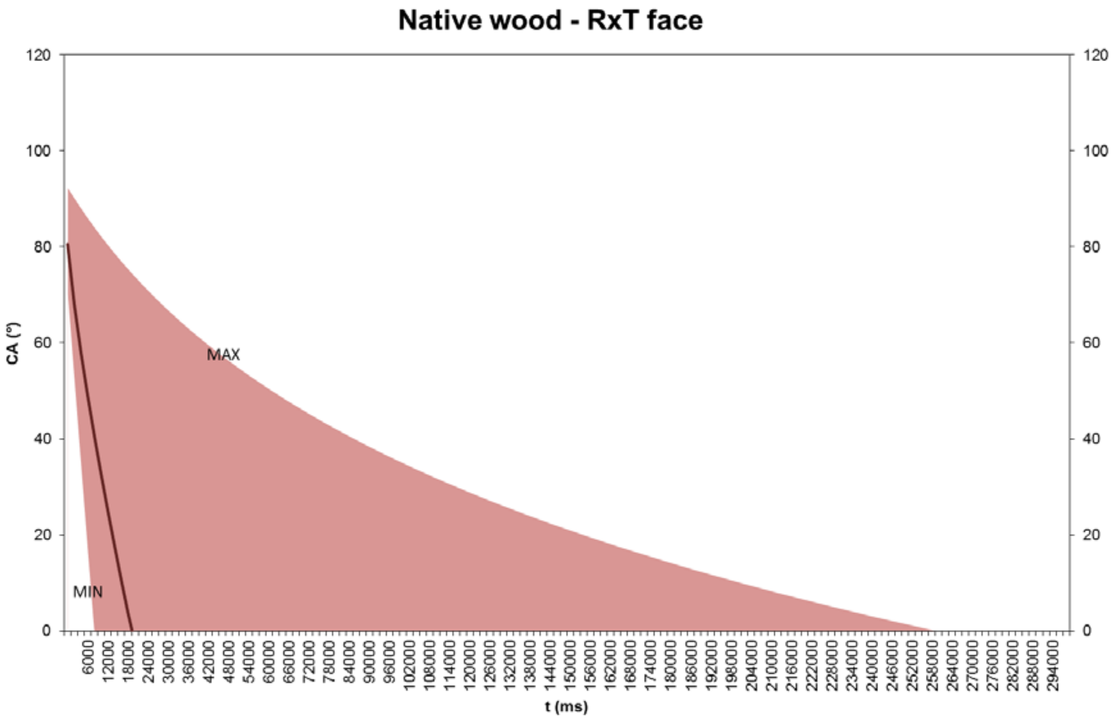

c)

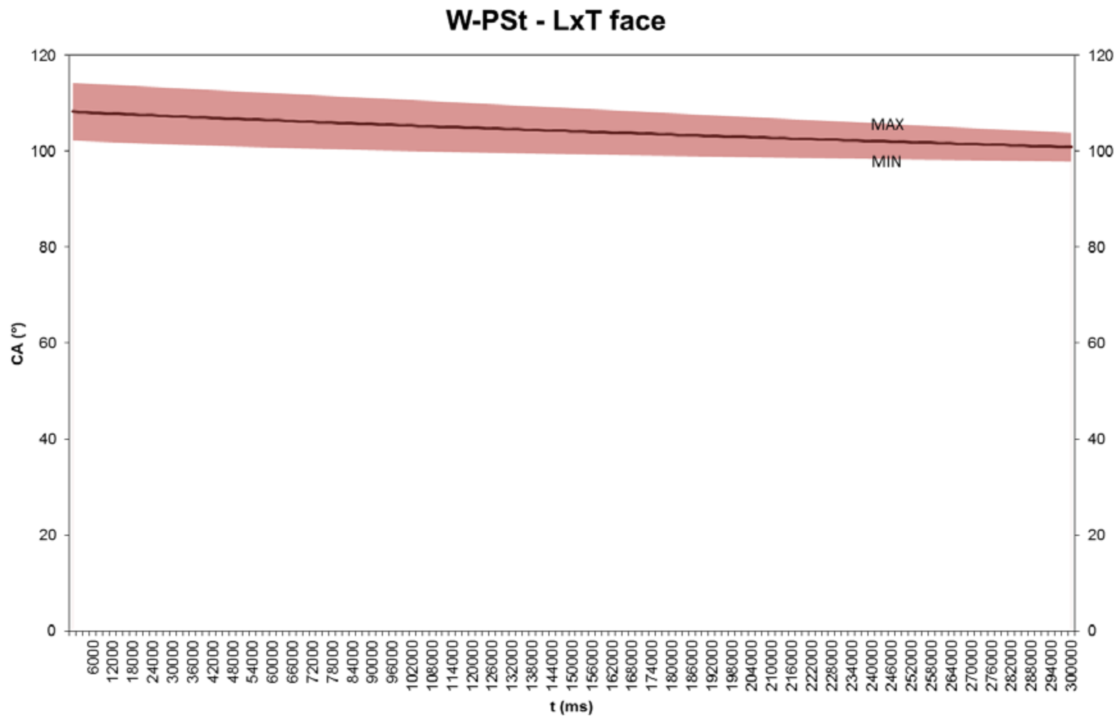

d)

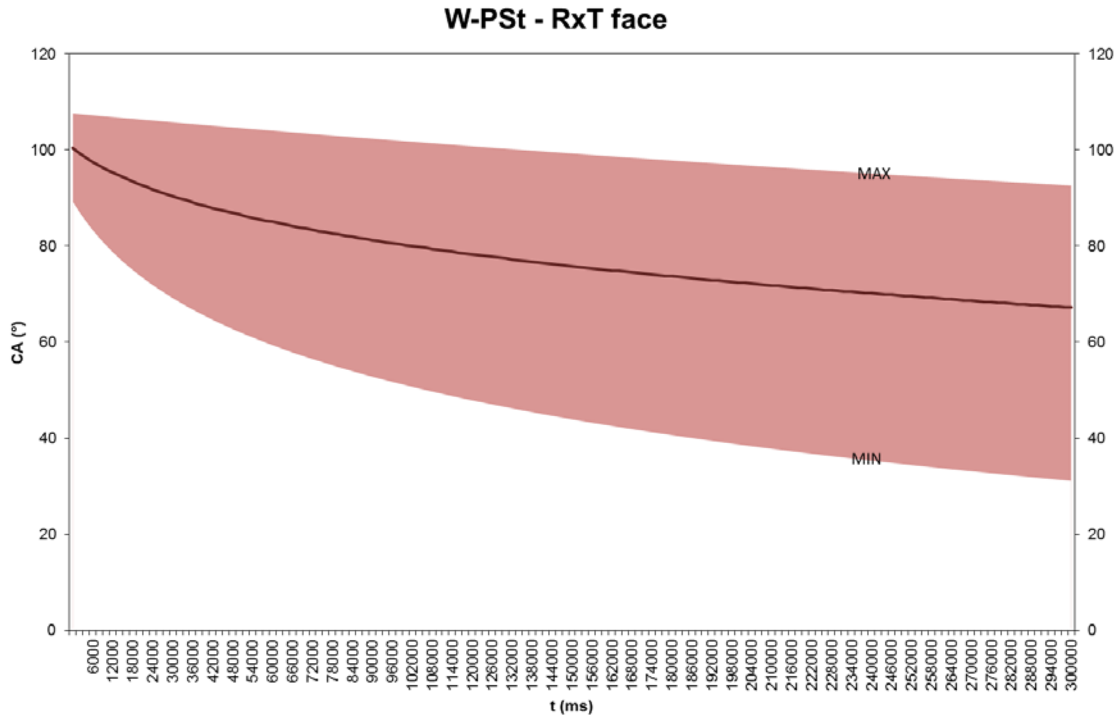

e)

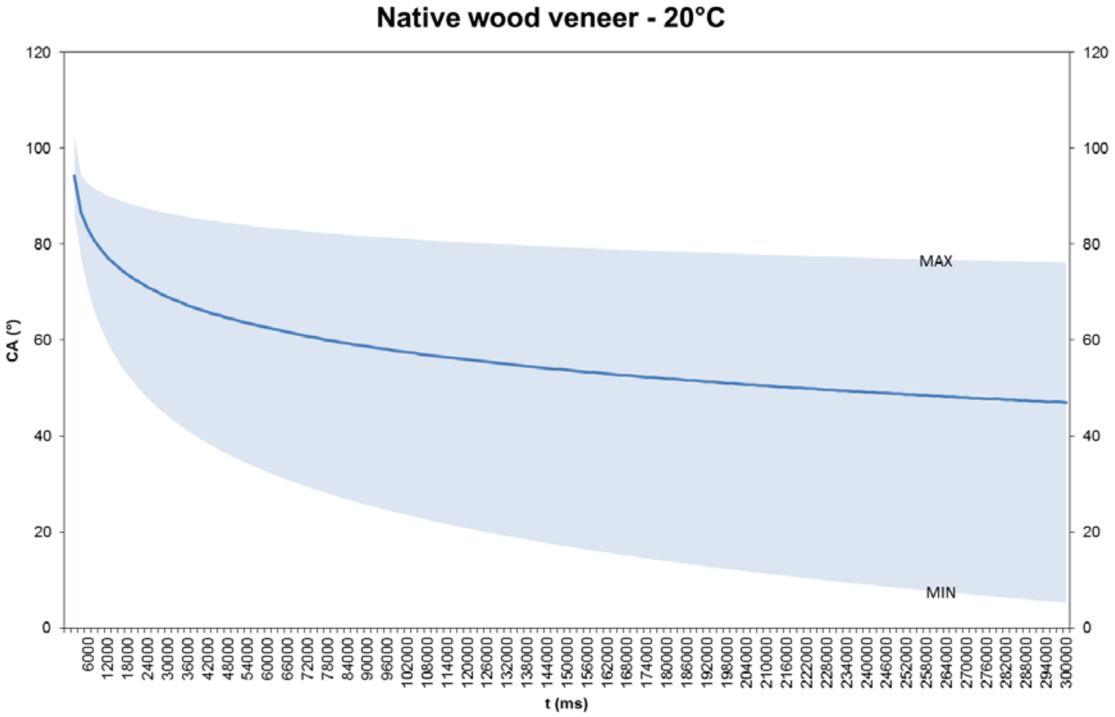
Native wood veneer - 20°C
CA (°)
t (ms)
MAX
MIN

f)

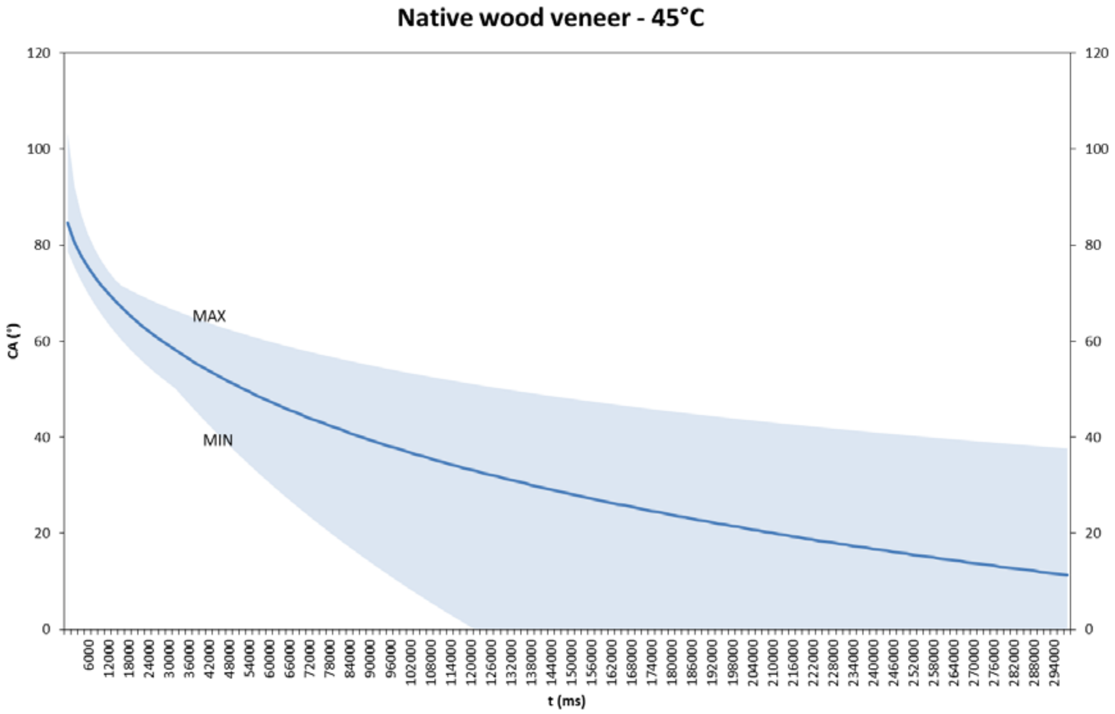
Native wood veneer - 45°C
CA (°)
t (ms)
MAX
MIN

g)

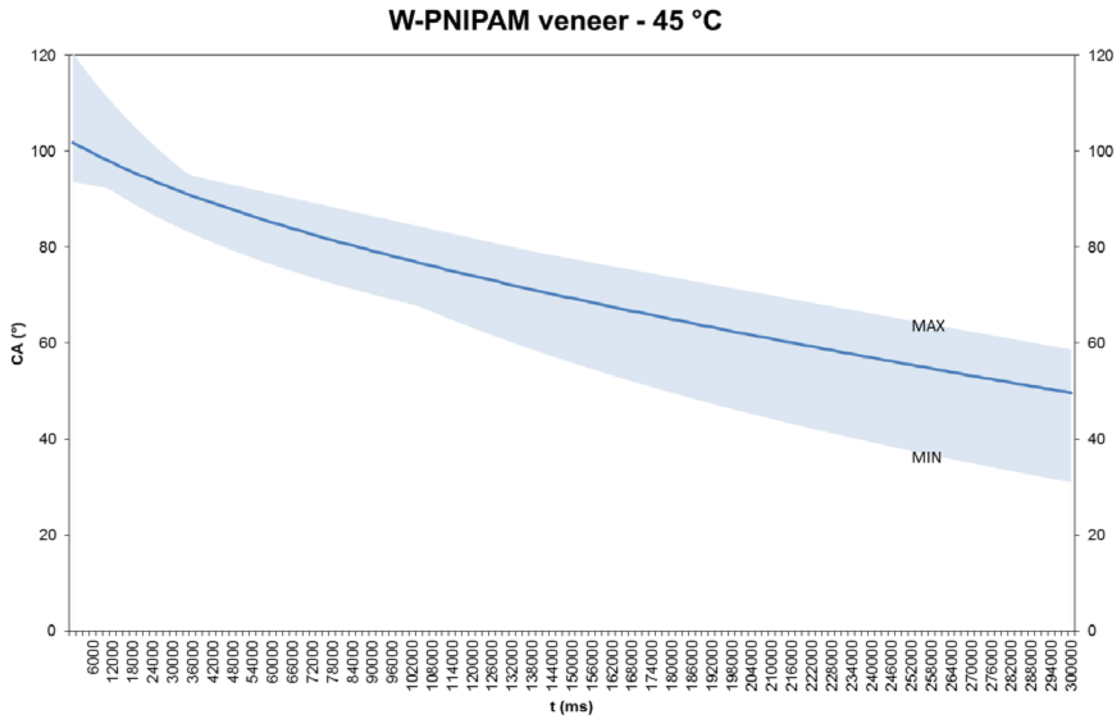

h)

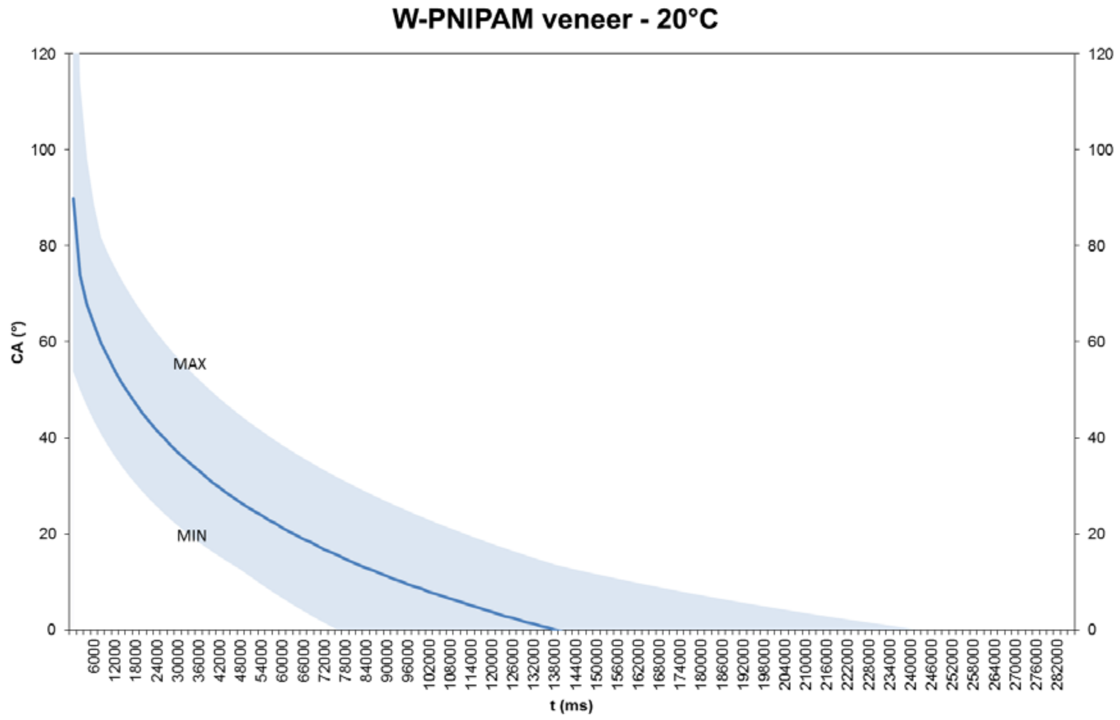

Figure S8: the plots show the CA evolution for a water drop on various surfaces averaged over 3 or 5 measurements, as well as the upper and lower values for these measurements. From a) to d) curves associated to measurements shown in Figure 4, and from e) to h), curves associated to measurements shown in Figure 5.

Dimensional stability study

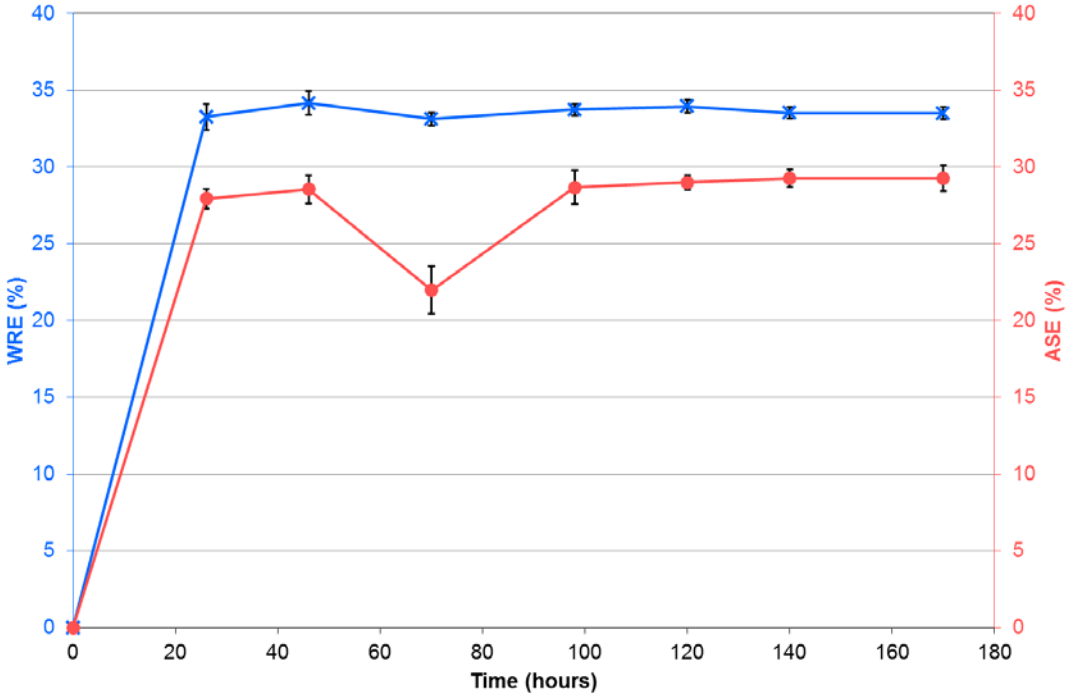

Figure S9: Plot showing the full water repellency and dimensional stability study on W-PSt samples, with the water-repellent effectiveness (WRE) and the anti-swelling efficiency (ASE). 10 cubes of W-PSt (with a total WPG of 27.3±0.3%) and 10 reference cubes were used for this experiment. Dry cubes are immersed in water, vacuum is drawn for 30 minutes, released for 1 hour, drawn again for 30 minutes and released over 24h. The new weight and dimensions are measured, and the cycles are repeated for a total of 7 days.

The total mass loss was calculated after 7 days in water (difference between the initial dry weight and the dry weight after 7 days), and was found to be 6.7±0.2%.

Analysis of leached material after 24h

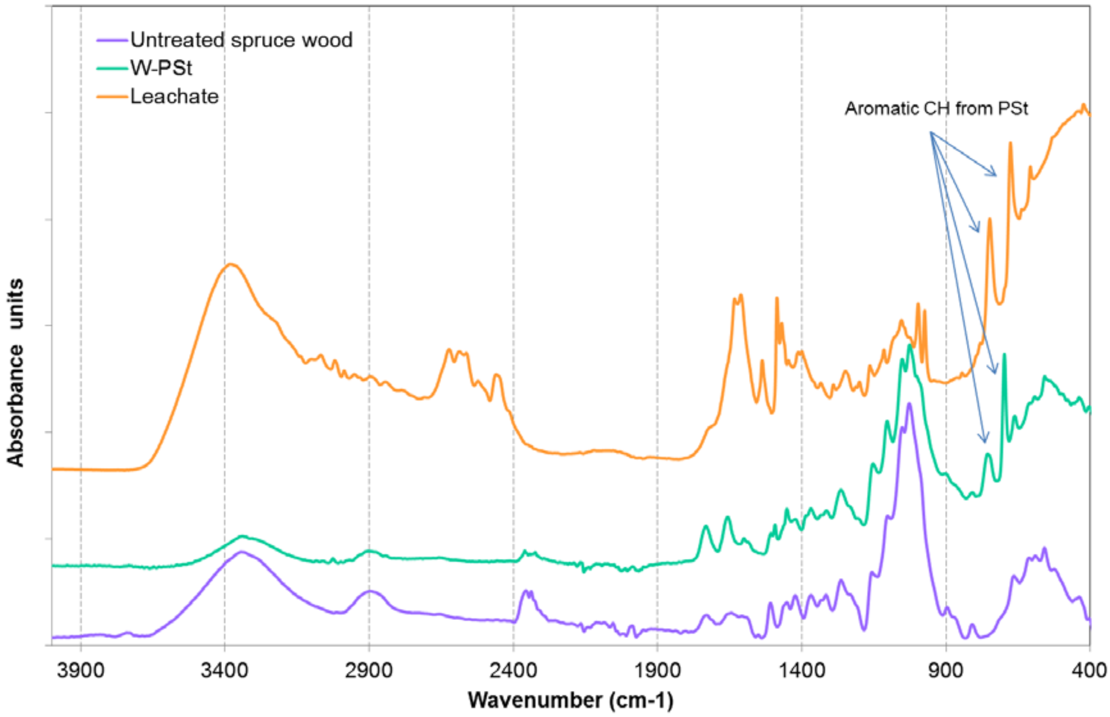

Figure S10: FTIR from material leached out after 24h of soaking in deionized water (leachate). The spectra for untreated and W-PSt are added for comparison. The presence of polystyrene chains in solution is clearly indicated by the peaks designated with arrows, around 650 and 750 cm^{-1}. No further leached material in measurable quantity was recovered after 48h.

References:

[1] R. M. Rowell, W. B. Banks, **1985**. Water repellency and dimensional stability of wood. Gen. Tech. Rep. FPL-50. Madison, WI: U.S. Department of Agriculture, Forest Service, Forest Products Laboratory; 1985: 24 pages
